# A rare presentation of empyema necessitatis

**DOI:** 10.4103/0970-2113.76311

**Published:** 2011

**Authors:** B. Mirza, L. Ijaz, A. Sheikh

**Affiliations:** *Department of Paediatric Surgery, The Children’s Hospital and The Institute of Child Health, Lahore, Pakistan E-mail: blmirza@yahoo.com*

Sir,

Empyema necessitatis is characterized by an extension of pus (empyema thoracic) from pleural cavities to the surrounding structures such as chest wall, mediastinum, pericardium, esophagus, retroperitoneum and so on.[1,2] A case of empyema necessitatis, presenting with cervical abscess, is being reported.

A 5-year-old boy was presented, in surgical emergency of our institution, with discharge of pus from a cervical lump during inspiration. There was a history of fever, cough and respiratory distress 10 days back, followed by the appearance of a lump in the right cervical region. The parents sought medical treatment from a local dispensary. A day back the lump spontaneously bursted with cupious amount of pus came out of the cervical lump.

The general physical examination revealed a temperature of 100°F, pulse 96/min, respiratory rate 33/min, and blood pressure within normal limits. The examination of the right cervical region showed an abscess cavity from which a small amount of pus was coming out during the inspiratory phase of the respiration. The respiratory system examination showed overt clinical signs of respiratory distress including nasal flaring, intercostal and subcostal retractions. The air-entry was reduced on the right side. A chest radiograph and ultrasound were requested that delineated empyema on the right side. The hemoglobin of the patient was 10g/dl with WBC count of 13000.

A tube thoracostomy with debridement of the cervical wound was performed under ketamin anesthesia. The patient was started on parenteral antibiotics including co-amoxiclav and amikacin, steam nebulization, and chest physiotherapy. The patient did well post-thoracostomy. The culture of the pus revealed *Staphylococcus aureus* which was sensitive to both of the drugs. The patient showed a commensurate amelioration of the symptoms. The chest drain was removed on tenth day of insertion and patient was discharged on oral co-amoxiclav for a week. The patient is symptom-free during a follow up of six months time.

Empyema thoracic is defined to have frank pus in the pleural cavity. Various kinds of complications can arise in untreated or partially treated patients. Empyema can extend to the surrounding structures; the reported sites are chest wall, peritoneum, pericardium, retroperitoneum, esophagus, mediastinum, abdominal wall, paravertebral space, vertebrae, bronchus, breast and diaphragm. The site in our case is very rare i.e. posterior triangle of the neck in the supraclavicular region. Empyema necessitatis usually presents with a lump in the chest along with clinical features of empyema thoracic such as fever, cough, respiratory distress etc. Rarely, the pus starts coming out from the wound,[1–3] but emission of pus from the lump during inspiration is a unique attribute in the index case. In our opinion, there should be a pleuro-cutaneous communication that ejects the pus from pleural cavity as the lungs expand during inspiration.

The common organisms isolated from the pus cultures in patients of empyema necessitatis are *Mycobacterium tuberculosis, Streptococcus pneumoniae, Staphylococcus aureus, Pseudomonas* and others. Rarely, methicillin-resistant Staphylococcus aureus (MRSA) has also been isolated from the pus cultures.[1,4] In our patient, the *Staphylococcus aureus* was sensitive to the empirical therapy instituted and patient showed a substantial improvement in the clinical condition and general well being.

Chest X-ray and ultrasound can give clues regarding empyema necessitatis but not in every case. An evidence of fluctuant lump along with radiographic and sonographic evidence of empyema thoracic raises a suspicion of empyema necessitatis. CT scan with contrast is highly sensitive in delineating the grade of empyema and its extension into the surrounding structures.[5]

The initial treatment of empyema necessitatis includes tube-thoracostomy with incision-drainage of the abscess cavity or debridement of the involved part and institution of parenteral broad spectrum antibiotics according to the culture and sensitivity. In few cases, thoracotomy and removal of the thick pleural cortex has to be performed to free the trapped lung.[1,5] Our case responded well to the tube-thoracostomy and antibiotics.

To conclude, empyema necessitatis is an entity with variable presentation. It has an excellent outcome when diagnosed and managed in time. An evidence of lump in the vicinity of chest along with clinical and plain radiographic signs of empyema thoracic is highly diagnostic of empyema necessitatis.
